# National Preparedness Month — September 2014

**Published:** 2014-09-05

Throughout September, approximately 3,000 organizations will participate in activities in support of National Preparedness Month. CDC supports this initiative by partnering with national, regional, state, and local government agencies, as well as private and public organizations, to encourage persons to take part in preparedness efforts at home, school, and throughout their communities.

For Preparedness Month 2014, CDC’s Office of Public Health Preparedness and Response has focused its efforts on developing messages and products designed to meet the needs of vulnerable populations (1). Vulnerable populations, those populations defined by economic disadvantage, language and literacy differences, medical issues and disability (physical, mental, cognitive, or sensory), isolation (cultural, geographic, or social), and age, have unique needs in a disaster or public health emergency. Using various tools (2) and workbooks (3), CDC is working to educate and empower all populations to make the right choices for their health and safety.

The unpredictable nature of disasters makes personal preparedness a necessity. In the case of vulnerable populations, there are unique considerations that must be taken into account when preparing for emergencies (4,5). Additional information about emergency preparedness and response is available at http://www.cdc.gov/phpr.
